# Examining the interglacial high‐elevation refugia scenario in East Asian subtropical mountain systems with the frog species *Leptobrachium liui*

**DOI:** 10.1002/ece3.4449

**Published:** 2018-08-24

**Authors:** Yuchi Zheng, Junhua Hu, Xiaomao Zeng

**Affiliations:** ^1^ Department of Herpetology Chengdu Institute of Biology Chinese Academy of Sciences Chengdu China

**Keywords:** cold‐adapted species, dispersal, East Asia, glacial cycles, interglacial refugia, subtropical mountains

## Abstract

The effects of Quaternary climatic oscillations on the distributions of organisms in different parts of the world are not equally well understood, limiting the ability to understand the determinants of biodiversity. Compared with the mountain regions in southern Europe and southwestern North America, such effects on high‐elevation species in the East Asian subtropical mountain systems located in southern and southeastern China have seldom been addressed. In this study, using *Leptobrachium liui* (Megophryidae), we made one of the earliest attempts to examine the interglacial high‐elevation refugia scenario in these Asian mountains. Based on our current understanding of the study system, we formulated a hypothesis that these frogs of western origin were distributed more widely and continuously during glacial phases, allowing eastward dispersal, and that they are currently isolated in interglacial refugia at higher elevations. Microsatellite data and mitochondrial and nuclear sequence data were obtained with extensive sampling followed by the synthesis of phylogeographic and population genetic analyses and modeling of the species distribution. The analyses revealed a sequential eastward divergence of microsatellite clusters and gene lineages accompanied by a decline in genetic diversity. Molecular dating estimates revealed divergence events in the Pleistocene, and a reduction in local populations was inferred to have occurred at a time comparable to the end of the last glacial. Strong genetic isolation by distance reflecting a more continuous historical distribution was detected. Furthermore, environmental niche models inferred a wide planar distribution during the last glacial maximum, providing further support for the hypothesis.

## 1 INTRODUCTION

Quaternary climatic oscillations affected the distribution of many species that have various environmental requirements (Hewitt, 2004; Hofreiter & Stewart, 2009). Such effects in different regions are not equally well understood, limiting the determination of biodiversity patterns. In general, the geographic ranges of organisms have changed cyclically according to glacial–interglacial cycles, with individual responses of species in space and time (Bennett & Provan, 2008; Stewart, Lister, Barnes, & Dalén, 2010). The distributions of cold‐adapted taxa are expected to be more widespread and continuous during glacial periods. Evidence consistent with this perspective has been reported from various regions, particularly from the mountain areas of Europe and North America (e.g., Djamali et al., 2012; Stewart et al., 2010; Wood, Vandergast, Lemos Espinal, Fisher, & Holycross, 2011; Woodruff, 2010). The East Asian mainland differs from Europe and North America by having remained largely unglaciated during the Pleistocene (Feng, Mao, Sandel, Swenson, & Svenning, 2016; Hewitt, 2000; Li, Shu, Zhou, Zhao, & Zhang, 2004). Relevant studies in this region are rare and have primarily focused on southwestern China and adjacent areas with their complex topography and high mountains, such as the Himalayas (e.g., Gao, Zhang, Gao, & Zhu, 2015; He & Jiang, 2014).

**Figure 1:**
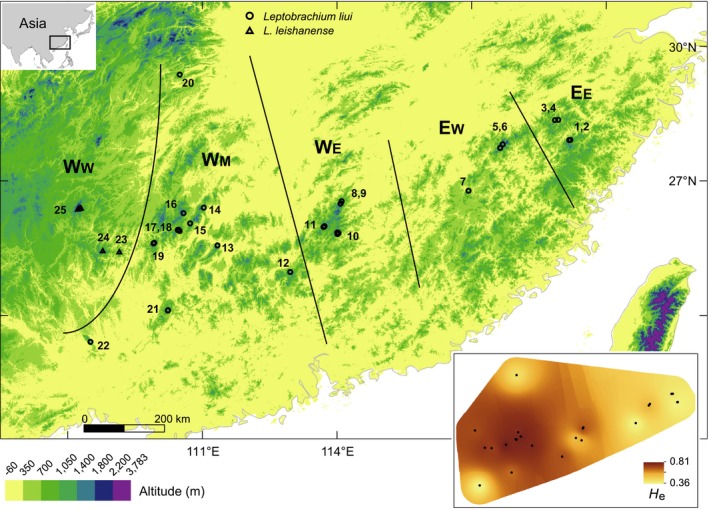
Sampling sites and geographic pattern of expected heterozygosity (*H*_e_) inferred from the 8‐loci microsatellite data. Sampling sites were grouped into five distribution areas, which were then grouped into two ranges, west (W) and east (E). W_W_: the most western area in which *L. leishanense* occurred; W_M_: middle W; W_E_: eastern W; E_W_: western E; E_E_: eastern E

East of these areas, numerous subtropical mountains stretch across southern and southeastern China. Their most recent rapid uplifts have been dated to before the Quaternary or during the early part [2.6–0.8 million years ago (Ma)] of the Quaternary (Guo, 1998; Zhou & Ren, 1984). With the highest peaks reaching ~2,100 m above sea level, these mountains are surrounded by lowlands ~200–400 m in elevation and exhibit strong temperature and habitat gradients (Figure 1). The limited available evidence suggests that certain cold‐adapted species ranged widely during glacial stages but became isolated at high elevations in these mountains during the present interglacial. First, pollen fossils of some current upland taxa were common at lower elevations during the last glacial maximum (LGM) (Zheng, 2000). Second, using mitochondrial DNA data, Tian, López‐Pujol, Wang, Ge, and Zhang (2010) identified several interglacial montane refugia for the pine species *Pinus kwangtungensis* and noted another three plant species with similar distributions and genetic patterns (but see Qiu, Fu, & Comes, 2011). Last, using molecular dating, Wu, Wang, Jiang, and Hanken (2013) found that all species of the endemic salamander genus *Pachytriton* had diverged before the Quaternary, and they reported that these cold‐adapted animals are currently restricted to high‐elevation refugia. Many other taxa that currently live at high elevations in these mountains may have experienced similar evolutionary histories.

**Figure 2:**
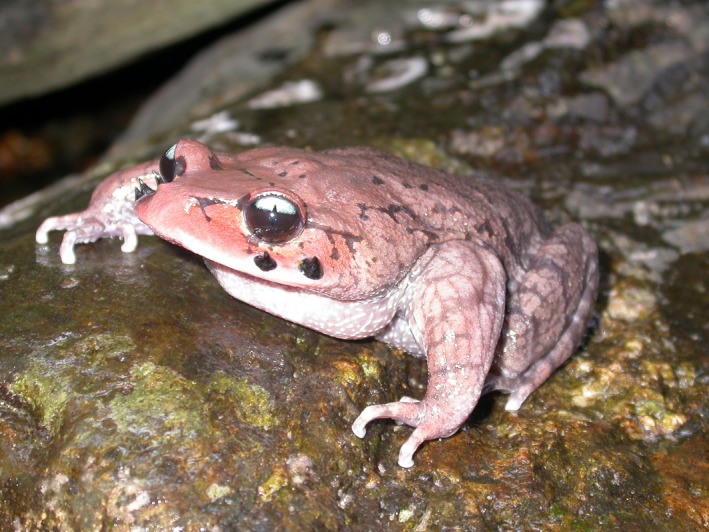
Male *Leptobrachium liui* with maxillary nuptial spines in Huaping Reserve, Guangxi, China. Photograph credit: Yuchi Zheng

Pope's spiny toad, *Leptobrachium liui* (Pope, 1947) (Figure 2), is ideal for evaluating this possibility. *Leptobrachium liui* is restricted to habitats at middle to high elevations (700–1,600 m) and is found in several major southern and southeastern China mountain ranges that are separated by low elevations or connected only by middle‐elevation passes and that form the northeastern range limit of the genus. This distribution may have evolved via west‐to‐east dispersal followed by long‐term occupation, as suggested by the deep eastward divergences among regional mitochondrial lineages (Kropf, Kadereit, & Comes, 2002; Waters, Fraser, & Hewitt, 2013; Zheng, Li, & Fu, 2008). In a much smaller range west of and adjacent to the distribution of *L. liui*, its sister species *L. leishanense* is restricted to similar habitats at 1,050–1,800 m in elevation (Zheng et al., 2008). These frogs breed in cold permanent streams from October to December, the hatching period extends over the entire winter (Li et al., 2009), and the tadpoles usually require 3 years to reach metamorphosis. Two factors suggest that the current disjunct distributions of these frogs were not caused by human activities (Sanderson et al., 2002), which are more intensive at low elevations. First, certain distribution localities were identified by systematic herptile surveys from low‐to‐high elevations conducted no later than the early 1960s (e.g., Liu & Hu, 1962) during a time when pollution was not a significant issue in the region. Second, no low‐elevation populations have been recorded in reserves providing natural habitats for *L. liui* or *L. leishanense*.

There is an ambiguous species boundary between the two taxa. The only morphological difference between the species is an internal subgular vocal sac in male *L. liui* that is not found in *L. leishanense*; however, both species call underwater (Zheng, Rao, Murphy, & Zeng, 2011), and intraspecific variation in the vocal sac condition has been reported in certain anurans (Che et al., 2009; Yu, Zhang, & Yang, 2010). Currently, adequate molecular evidence distinguishing these two species is lacking. In a nuclear gene tree created by Zheng et al. (2008), most haplotypes of *L. liui* are regionally endemic, while *L. leishanense* shares its haplotypes with western *L. liui*. In a mitochondrial gene tree created by Matsui et al. (2010), *L. leishanense* is nested within lineages recognized as *L. liui* in Zheng et al. (2008) and Frost (2018). Therefore, *L. leishanense* may be *L. liui* populations occupying the western edge and a possibly ancestral range of the generally east–west distribution, and including *L. leishanense* is imperative when testing a hypothesis on the historical distribution of *L. liui*.

The interglacial high‐elevation refugia scenario includes a contraction after the end of the last glacial ~0.01 Ma, which is expected to have caused a reduction in population size. Rapidly evolving and hence highly polymorphic microsatellites may provide adequate data to assess whether there has been a recent decline in population size (Peery et al., 2012). Approximate Bayesian computation (Beaumont, 2010) estimates the parameters and relative posterior probabilities for a set of user‐specified demographic models, which can be complex (e.g., >1 population size changes). Range expansions of limited extent might have occurred in some cold‐adapted species after the warmest period of the present interglacial, the Holocene climatic optimum (Bell et al., 2010), and resulted in detectable demographic expansions. A model defining an increase after a decline in population size can be used to account for this possibility. In southern China, the optimum was dated to approximately 10,000–6,000 years ago, and it was followed by a decrease in the extent of forests (Zhou et al., 2004). Another possible demographic change that may complicate the inference is a more recent decline, within hundreds of years, caused by human impact. This can be considered at first by a simple model of one change/decline in population size at a time to be estimated. With immediate effects on microsatellite variation and limited recovery time, the sign of a newly occurred reduction would be readily detected (Cornuet & Luikart, 1996; Garza & Williamson, 2001; Hoban, Gaggiotti, & Bertorelle, 2013).

Under the interglacial refugia scenario, *L. liui* may exhibit an isolation by distance (IBD) pattern of genetic structure across the range. The dispersal abilities of amphibians are generally limited (Cushman, 2006). During glacial cycles, glacial phases of ~0.1 million years are normally an order of magnitude longer than interglacial phases (Sigman, Hain, & Haug, 2010; Stewart et al., 2010; Woodruff, 2010). If these frogs ranged more widely and continuously during the glacial phases, their distribution over the long term should have been more continuous, with gene flow interrupted only by short interglacial disjunction into high elevations (Schmitt, 2007). Change in the genetic differentiation between populations often requires long time periods (Ibrahim, Nichols, & Hewitt, 1996; Leng & Zhang, 2011; Palo et al., 2004). It is unlikely that an IBD pattern established over long periods of a continuous distribution (Slatkin, 1993; Wright, 1943) would have been erased by the increased genetic differentiation during the current interglacial of ~0.01 million years (Mastretta‐Yanes et al., 2018). In theory, the effect of such increased genetic differentiation on the Mantel correlation test for IBD may be analogous to adding a constant to the genetic‐distance variable, which will not affect the detection of a correlation. If an IBD pattern is detected, the genetic distances are expected to be better predicted by log‐transformed geographic distances (two‐dimensional IBD) than by raw geographic distances (one‐dimensional IBD) when the habitat is more planar (Rousset, 1997) over the long term.

The predicted planar distribution during glacial stages can also be examined by correlative species distribution modeling. Specifically, ecological niche models are used to generate the distribution projection based on reconstructed LGM climatic variables. Different variables reconstructed using different models can result in dissimilar and even contradictory projections with respect to a given question of interest. In such situations, molecular evidence can be useful in interpreting the projections (Gavin et al., 2014).

In this study, using extensive sampling of both species with 907 individuals from 25 sites, 14 microsatellite loci, one mitochondrial locus, and one nuclear locus, we tested a hypothesis integrating eastward dispersal with the interglacial high‐elevation refugia scenario. We hypothesised that *L. liui* originally occurred in the west, and spread from high‐elevation refugia to a more continuous and planar habitat during Quaternary glacial periods that allowed dispersals extending the range of distribution eastward to the current one. We made six predictions: (a) sequential eastward divergence of microsatellite clusters and gene lineages; (b) molecular date estimates of the Pleistocene for at least the more recent divergences; (c) west‐to‐east decline in genetic variation consistent with the loss of diversity during dispersal; (d) an estimated reduction in local populations at a time comparable to the end of the last glacial (~0.01 Ma); (e) two‐dimensional IBD fitting the genetic structure better than one‐dimensional IBD if an IBD pattern was found; and (f) a wider and more continuous distribution during the LGM inferred by correlative species distribution modeling. We examined these predictions based on the combined data of *L. liui* and *L. leishanense* because we detected lower genetic differentiation between *L. leishanense* and nearby *L. liui* than between the latter and the other populations of *L. liui*, suggesting that *L. leishanense* is a synonym of *L. liui*.

## 2 MATERIALS AND METHODS

### 2.1 Sampling and molecular data

A total of 799 individuals from 22 sampling sites of *L. liui* and 108 individuals from three sites of *L. leishanense* were included as the ingroup, and the sampling spanned the distributions of both species (Figure 1). Among them, 832 individuals were included in the genotyping efforts for 14 microsatellite loci; 880 individuals were sequenced for a mitochondrial locus; and a representative subset of 101 individuals was sequenced and haplotyped for a nuclear locus.

The microsatellite loci included VIB‐B4, VIB‐C10, VIB‐D5, CHA1, CHA6, CHA9, LSMT6, LSMT8, CHA4, CHA7, CHA8, CHA11, LSMT4, and LSMT9 (Bi, Deng, Crosby, & Fu, 2010; Hu, Xia, Zheng, & Zeng, 2012; Wang et al., 2011). The PCR fragments containing primers fluorescently labeled with FAM, TAMRA, or HEX were evaluated along with the GS500 size standard using an ABI 3730 analyzer (Applied Biosystems, Foster City, CA, USA). The raw data were visualized, and the alleles were manually scored using GeneMarker 1.95 (SoftGenetics, State College, PA, USA). Tests for the presence of stuttering, allele dropout, and null alleles were performed using Micro‐Checker 2.2.3 (van Oosterhout, Hutchinson, Wills, & Shipley, 2004). An exact test of Hardy–Weinberg equilibrium and a likelihood ratio test for linkage disequilibrium were conducted in GENEPOP 4.2 (Rousset, 2008). The 745‐bp mitochondrial *nad1* locus consisted of regions of the tRNA‐Leu (16 bp) and NADH dehydrogenase subunit 1 genes; the nuclear *rag1* locus contained an 800‐bp partial sequence of the recombinase activating protein 1 gene. The primers used in the PCR and Sanger sequencing (ABI 3730) experiments included Leu‐1L, Leu‐5L, Rag1‐3L, Rag1‐2H, Rag1‐6H (Zheng et al., 2008), and a newly designed ND1‐6H (5′‐TgACCCTAAgAACAgAATTACTgATAAggT‐3′). The *rag1* PCR products were directly sequenced, and when two or more dimorphic sites resulting from heterozygosity were detected, 3 to 16 (mean 9.2) clones were sequenced to distinguish among the haplotypes.

Three congeners were used as outgroups for reconstructing gene trees. In the reported mitochondrial gene trees, *L. boringii* belonged to the clade (or one of the clades) most closely related to *L. liui* and *L. leishanense* (Matsui et al., 2010; Zheng et al., 2008). Thus, *L. boringii* was selected as the outgroup for the *nad1* data. In the reported *rag1* tree, the lineages of *L. liui* and *L. leishanense* did not coalesce until the most recent common ancestor of *L. ailaonicum*,* L. boringii*,* L. chapaense*,* L. leishanense*,* L. liui*, and *L. promustache* because of incomplete lineage sorting (Zheng et al., 2008). The sister group of this clade contained *L. ngoclinhense* and *L. xanthospilum*, which were used as outgroups for the *rag1* data. The *nad1* sequences of 29 individuals and the *rag1* sequences of 27 individuals were obtained from Zheng et al. (2008). Detailed specimen information, GenBank accession numbers, and haplotype designations are presented in Supporting Information Table S1.

Sequence alignments were performed with ClustalX 2.1 (Larkin et al., 2007) and checked against the amino acid sequences. The Bayesian information criterion implemented in jModelTest 0.1.1 (Posada, 2008) was used to select the nucleotide substitution model that best fit the data.

### 2.2 Microsatellite clustering, gene tree, and divergence date

The clustering method implemented in STRUCTURE 2.3.4 (Pritchard, Stephens, & Donnelly, 2000) was applied to the microsatellite data. The admixture ancestry model was adopted, with the value of the Dirichlet parameter α for the degree of admixture inferred from the data. The prior population information was not used. The correlated allele frequencies model was used, with the value of the allele frequencies parameter *λ* fixed to 1. The burnin length and the run length after burnin were set to 100,000 and 200,000 steps, respectively. The number of clusters (*K*) evaluated ranged from one to the number of sampling sites included plus one. For each *K*, 20 independent runs were conducted, and the 10 runs with the highest likelihoods were included in the follow‐up analyses. In STRUCTURE HARVESTER 0.6.94 (Earl & vonHoldt, 2012), the results were summarized and compared using the Evanno method and the Delta *K* (Evanno, Regnaut, & Goudet, 2005) was calculated. Combining runs with the same mode of clustering, a graphical representation of the *Q*‐matrix was generated using CLUMPAK (Kopelman, Mayzel, Jakobsson, Rosenberg, & Mayrose, 2015). The following four criteria were considered in detecting values of *K* that captured the major structures in the data: Delta *K*, a high rate of change of the likelihood, the most individuals from a sampling site being assigned to a population with high possibility (say ≥0.85), and a reasonable biological interpretation (Meirmans, 2015). When hierarchical clusters were obtained using the selected *K* values, they were summarized as a tree‐like population history.

Gene trees of the unlinked *nad1* and *rag1* loci were separately reconstructed because the relationships among haplotypes were of primary interest. Using the *nad1* data, divergence times were estimated simultaneously with Bayesian topology in BEAST 2.1.2 (Bouckaert et al., 2014). The monophyly of the ingroup was constrained. The uncorrelated lognormal model was used to describe the relaxed clock. The calibrated Yule model was specified for the tree prior. Suitable fossil calibrations were not available. An evolutionary rate of 0.69% ± 0.3% divergence per million years per lineage that is commonly used for the anuran *nad1* gene was used to calibrate the tree (e.g., Hoffman & Blouin, 2004; Macey et al., 1998; Sanguila, Siler, Diesmos, Nuneza, & Brown, 2011). The uncorrelated lognormal relaxed clock mean (ucldMean) was constrained between 0.0039 and 0.0099 substitutions per site per million years, and a normal distribution was applied (mean 0.0069, sigma 0.0015). For *rag1*, Bayesian inference was performed with MrBayes 3.2.1 (Ronquist & Huelsenbeck, 2003), using four chains. In both BEAST and MrBayes analyses, a run length of 20 million generations and a sampling frequency of 2,000 generations were applied, and four independent runs were performed to avoid local optima. The performance of the runs was assessed using Tracer 1.6 (Rambaut, Suchard, Xie, & Drummond, 2014) to ensure convergence and adequate effective sample sizes (>200; Drummond, Ho, Phillips, & Rambaut, 2006). Then, the last 90% of the samples for each of the four runs were combined to produce a maximum clade credibility tree.

### 2.3 Genetic diversity and structure

Diversity parameters were estimated for each local population, and the inverse distance‐weighted method incorporated in ArcGIS 9.2 (ESRI, Redlands, CA, USA) was used to present spatial patterns in diversities. For the microsatellite data, the expected heterozygosity was estimated using Arlequin 3.5.1.2 (Excoffier & Lischer, 2010), and allelic richness and private allelic richness were calculated with rarefaction to the smallest sample size of 8 using HP‐Rare 1.1 (Kalinowski, 2005). The haplotype diversity and average number of pairwise nucleotide differences were obtained from the *nad1* and *rag1* data using DnaSP 5.10.01 (Librado & Rozas, 2009).

Various measures of population differentiation were calculated and used for further analysis. For the microsatellite data, the *F*_ST_ value was estimated according to Weir (1996) using the software FreeNA (Chapuis & Estoup, 2007), applying the *ENA* method for correcting genotype data with null alleles, and the *D*_est_ value (Jost, 2008; Leng & Zhang, 2011) was estimated using SMOGD 1.2.5 (Crawford, 2010). For the *nad1* and *rag1* data, analysis of molecular variance (AMOVA) was conducted using Arlequin. To assess the major pattern of differentiation between regional population groups, principal coordinate analysis (PCoA) was conducted on individual interpopulation difference matrices using GenAlEx 6.502 (Peakall & Smouse, 2012), with negative values set to zero. The Isolation by Distance Web Service 3.23 (Jensen, Bohonak, & Kelley, 2005) was used to perform the Mantel test for correlations between the genetic (*F*_ST_, *D*_est_, or AMOVA *Φ*_ST_) and geographic distances, and 10,000 randomizations were applied, with the negative genetic distances set to zero. The geographic distances were calculated using coordinates and analyzed with and without logarithmic transformation.

### 2.4 Population size change

Addressing the predicted recent decline ~0.01 Ma in each local population, the approximate Bayesian computations were applied to the microsatellite data using DIYABC 2.1.0 (Cornuet et al., 2014). Five demographic scenarios were compared. In the one‐change scenario, a change in population size occurred sometime within 10–10,000 generations ago. Because the tadpoles of these frogs require 3 years to reach metamorphosis, a generation time of 4 or more years could be reasonably assumed. Consequently, the prior time extended to no <40,000 years ago. The following three scenarios were special cases of the one‐change scenario: Population growth occurred in the one‐growth scenario, no changes (rate = 0) in population size occurred in the constant‐size scenario, and a decline in population size occurred in the one‐decline scenario. Lastly, in the growth‐after‐decline scenario, two changes in population size occurred 10–10,000 generations ago; a decline first occurred from a population size larger than the current one, and then, an increase in population size occurred. This scenario was designed to model a decline after the LGM followed by growth after the Holocene climatic optimum, with the resulting current population remaining smaller than that at the LGM.

For all scenarios, the prior effective population size was set to 10–10,000, and uniform distributions were applied to the time and population size priors. All loci were included in a single locus group. With default priors valid for many species (Cornuet et al., 2015), a generalized stepwise mutation model allowing single‐nucleotide indels was applied to the data. All four summary statistics for one sample of microsatellite data were used. For each scenario, 10,000,000 simulations were performed to build the reference table. Among them, the 10,000 (0.1%) with summary statistics closest to the observed ones were used to estimate the historic demographic parameters. The relative posterior probabilities of different scenarios were computed using the direct estimate and logistic regression approaches with 500 and 10,000 simulated datasets closest to the observed dataset, respectively (Beaumont, 2010). For analyses evaluating confidence in the scenario choice, the posterior‐based error computation was adopted. Computations were processed on 1000 simulated pseudo‐observed test datasets, each of which drew (with replacement) the scenario ID and parameter values from the 500 simulated datasets closest to the observed one. Then, the above procedure for computing posterior probabilities of scenarios was conducted on each pseudo‐observed test dataset, and the right scenario and the scenario having the highest probability were reported.

### 2.5 Species distribution modeling

The LGM and current species distributions were modeled for comparison. The analyses were conducted at a spatial extent of 21.25–31°N and 106.5–122.25°E. This area did not extend substantially from the ranges of the study species and the mountains (Figure 1). The current range of the species was expected to be indicative of its LGM distribution, which was fragmented into various present‐day local refugia according to our hypothesis. Ecological niche modeling was conducted using Maxent 3.3.3k (Phillips, Anderson, & Schapire, 2006). The Maxent model, which works by optimizing a set of constraints representing the incomplete information about distribution and evaluating the environmental suitability of each grid cell within the study area (Elith et al., 2011; Phillips et al., 2006), has been shown to have good predictive performance across various applications (Elith et al., 2006). Nineteen bioclimatic variables were obtained from the WorldClim database (Hijmans, Cameron, Parra, Jones, & Jarvis, 2005), with a resolution of 2.5 arc‐minutes. Georeferenced occurrence records were obtained from our field surveys, and only one occurrence per grid cell was ensured. The final compilation included 29 records from both taxa (Supporting Information Table S2). There is a need for variables to be as proximal as possible, and over‐fitting could occur if there are too many strongly collinear variables, especially with the small sample size of the present records (Qiao, Peterson, Ji, & Hu, 2017; Synes & Osborne, 2011). We hence conducted a Pearson's correlation test between variables (Dormann et al., 2013) and a jackknife analysis of variable importance to the Maxent model (Phillips et al., 2006). Two criteria were considered for choosing between highly correlated temperature/precipitation variable pairs (|*r*| > 0.8), namely, larger contribution to model development and putative greater biological importance. As a result, 10 variables were retained as follows: annual mean temperature, mean monthly temperature range, annual temperature range, mean temperature of the wettest quarter, mean temperature of the warmest quarter, mean temperature of the coldest quarter, annual precipitation, precipitation of the driest month, precipitation seasonality, and precipitation of the warmest quarter.

In the Maxent analysis, we mainly used the default settings and ran models with 10 bootstrap replicates by randomly assigning localities to the training (75%) and test (25%) data sets. The default settings have been validated over a wide range of species using different sets of environmental variables in various regions of the world and have been shown to achieve good performance (Phillips & Dudík, 2008). The replicates from Maxent were used as proxy models to develop the consensus‐based ensemble forecasting (Araújo & New, 2007), and the mean suitability of the replicate outputs was calculated using ArcGIS 9.2 (ESRI). The easily interpretable logistic output format was selected with suitability values ranging from 0 (lowest) to 1 (highest) (Phillips & Dudík, 2008). Model performance was assessed using the average AUC (area under the receiver operating curve) score. The most important variables to the model were identified by their percent contribution values and the results of the jackknife tests (Phillips, 2017). How they affected the distribution was interpreted with the aid of the response curves made using only the corresponding variable. The paleodistributions in the LGM were inferred with the same resolution of 2.5 arc‐minutes (Hijmans et al., 2005). The climatic variables of the LGM reconstructed by the community climate system model (CCSM; Collins et al., 2006) and the model for interdisciplinary research on climate (MIROC; Hasumi & Emori, 2004) were used. For each important variable, the LGM reconstructions and current estimates were compared to characterize the distribution and extent of possible novel climate conditions, a potential issue of model projecting (Phillips, 2017).

## 3 RESULTS

### 3.1 Molecular data

Eight microsatellite loci, VIB‐B4, VIB‐C10, VIB‐D5, CHA1, CHA6, CHA9, LSMT6, and LSMT8, were successfully genotyped for all sampling sites included, forming the 8‐loci data used for interpopulation analyses and comparisons. From the 14‐loci data with missing genotypes, Micro‐Checker did not return evidence of stuttering or allele dropout and detected 13 cases (4.8%) of potential null alleles (all *p *<* *0.05), with one to three cases per locus. After sequential Bonferroni corrections, four cases (1.5%) of significant (0.05 level) deviation from Hardy–Weinberg equilibrium were identified in three populations, and 31 departures (2.7%) from linkage equilibrium (one or two cases per locus pair) were significant at the 0.05 level.

The *nad1* dataset had 122 haplotypes and 745 nucleotide sites of which 184 sites were variable and 138 were parsimony‐informative among the ingroup members. Among the 138 sites, 0, 19, and 2 sites belonged to the tRNA‐Leu gene, the 1st codon position, and the 2nd codon position, respectively. The *rag1* dataset had 43 haplotypes and 800 sites, of which 33 were variable and 16 were parsimony‐informative among the ingroup members. Of those 16 sites, 2 and 3 were from the 1st and 2nd codon positions, respectively. To avoid potential overparameterization of the intraspecific analysis, we did not divide the *nad1* and *rag1* datasets into partitions. For gene tree reconstruction, the TrN+G model was selected for the *nad1* data, and the K80 + I model was selected for the *rag1* data.

### 3.2 Microsatellite clusters, gene trees, and divergence dates

**Figure 3:**
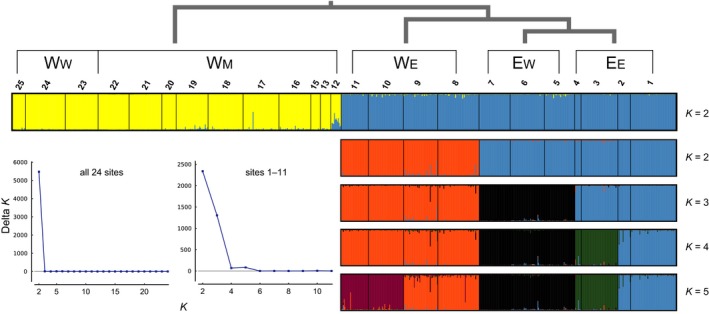
Bayesian clustering for all samples (*K *=* *2) and for the individuals from sites 1–11 (*K *=* *2 to *K *=* *5) based on the 8‐loci data. High Delta *K* values were obtained for these *K* priors. Each individual is represented as a vertical bar colored in proportion to its estimated membership in each cluster. The top phylogenetic hypothesis of clusters corresponding with individual distribution areas was summarized from these results

The STRUCTURE clustering analysis detected sequential eastward divergences and grouped *L. leishanense* (W_W_) with nearby *L. liui* (W_M_) (Figures 1 and 3; Supporting Information Table S3). Using the 8‐loci dataset containing all sampling sites, *K *=* *2 was selected (mean α = 0.031). The samples from areas W_W_ and W_M_ and those from W_E_ and E were separately assigned to two groups, which were then analyzed individually. Admixture was observed in the intermediate locality 12. Within the W_W_ + W_M_ group, interpretations of the clustering results were challenging. For example, five clustering modes were found among the 20 runs when *K *=* *2. The mode with the lowest likelihood divided W_W_ from W_M_, whereas the mode with the highest likelihood clustered one northern and two southern insular sites (20–22) as a single population. Relevant to these results, a population structure driven by IBD complicated the STRUCTURE clustering analysis (Pritchard, Wen, & Falush, 2010). Within the W_E_+E group, a tree‐like population history was inferred. For *K *=* *2, the individuals from E were separated from those from W_E_. For *K *=* *3, the individuals from E_E_ were further separated from those of E_W_. When *K *=* *4, within E_E_, the individuals of sites 1 and 2 were separated from those of sites 3 and 4.

**Figure 4:**
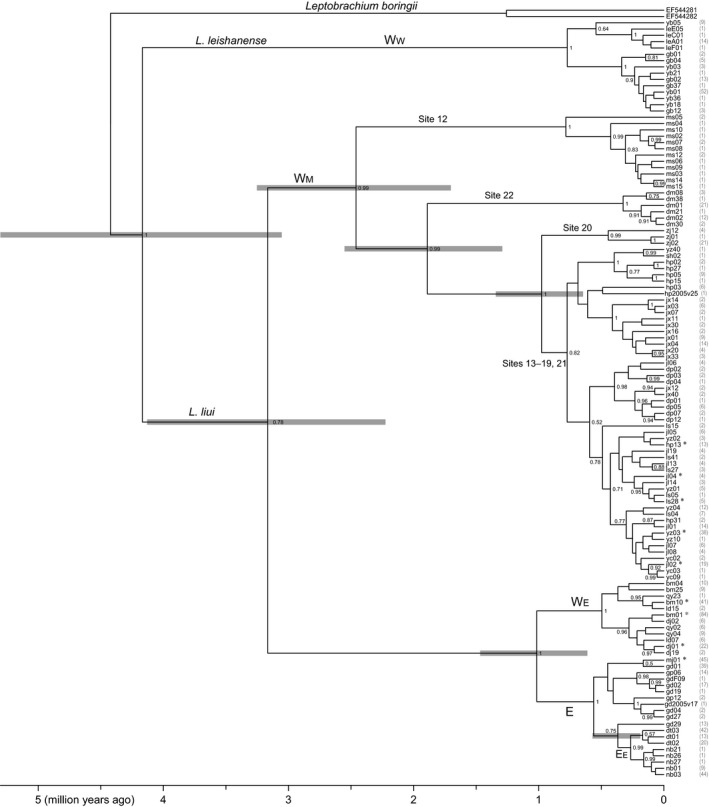
Time‐calibrated *nad1* gene tree of *L. liui* and *L. leishanense*. The gray bars indicate the 95% highest posterior densities for the age estimates. Numbers next to nodes are Bayesian posterior probabilities ≥ 0.50. Values in parentheses are haplotype frequencies. The haplotypes marked with an asterisk are shared by populations close to one another. Names of the distribution regions and sampling sites correspond with those in Figure 1

Sequential eastward divergences that occurred approximately 3.2–0.4 Ma and, more generally, strong and long‐standing geographic structures were revealed by the *nad1* time‐calibrated tree (Figure 4). The *nad1* tree exhibited mostly well‐supported major lineages consistent with the mitochondrial topology in Zheng et al. (2008) with fewer localities but additional genes (2,577 sites). It could be summarized as (W_W_,(W_M_,(W_E_,E))), with a 1.5–0.6 Ma divergence date between W_E_ and E. In E, the E_E_ lineage was nested within the E_W_ lineages and was estimated to have diverged from the latter 0.6–0.2 Ma. The distribution could be divided into eight areas/subareas (W_W_, four W_M_ subareas, W_E_, E_W_, and E_E_) that correspond exclusively to various genetic lineages that diverged approximately 4.2–0.4 Ma (Figures 1 and 4). Within the areas/subareas, each main subclade was formed by all haplotypes from one or a few adjacent local populations, and only populations close to one another shared haplotypes.

The *rag1* tree showed eastward divergences and close relationships between W_W_ and W_M_ haplotypes (Supporting Information Figure S1). Four haplotypes were observed in the eastern range E, one in E_E_ and four in E_W_. They were most closely related to those found in the nearby western populations 8–11 (W_E_) and 12, forming two separate lineages. These and another two W_E_ lineages were nested within several W_M_ and W_W_ lineages.

### 3.3 Genetic diversity and structure

A general pattern of a decline in genetic diversity was detected moving eastward along the distribution, with low levels of genetic diversity found in both the most southern (22) and northern (20) sites (Supporting Information Table S4). The spatial pattern of the expected heterozygosity for the microsatellite data is presented in Figure 1 as an example.

**Figure 5:**
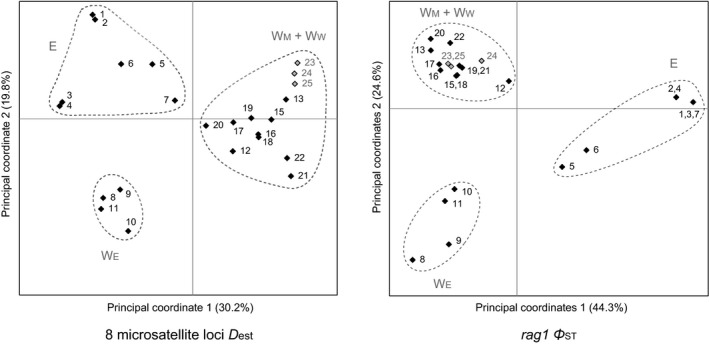
Principal coordinates analyses of local populations based on *D*_est_ (8 microsatellite loci) and *Φ*_ST_ (*rag1*) estimates. Populations were grouped according to their distribution areas

The close relationship between W_W_ and W_M_ populations was also revealed by PCoA and AMOVA of the microsatellite and *rag1* data. The samples from W_M_ + W_W_, W_E_, and E were easily distinguished in the PCoA plots, and the W_W_ points were mixed with or located very close to the W_M_ points (Figure 5 and Supporting Information Figure S2). The *rag1* AMOVA *Φ*_CT_ estimates were 0.370 (*p *<* *0.05) for the group pair W_M_ and W_E_ + E, 0.047 (*p *=* *0.198) for W_M_ and W_W_, and 0.422 (*p *<* *0.05) for W_W_ and W_E_ + E. These lines of comparison had not been applied to the *nad1* data, in which the gene tree showed a sister‐group relationship between W_W_ and the others.

A strong two‐dimensional IBD model fitted the genetic variation better than the one‐dimensional IBD model across the range. Genetic variation was explicitly spatially structured and low levels of differentiation were limited to local scales (Supporting Information Table S5). For the microsatellite data, the mean *F*_ST_ and *D*_est_ estimates were 0.31 and 0.63, respectively. For *rag1* and *nad1*, as revealed by AMOVA, the among‐population variation accounted for 54.7% and 95.2% (both *p *<* *0.05) of the total variation, respectively. These genetic variations could be primarily explained by logarithmic geographic distances, which consistently performed better than the linear geographic distances (Table 1).

**Table 1:** Estimated *R* ^2^ of the Mantel test for the correlation between geographic distances (GD) and genetic distances for *L. liui* + *L. leishanense* (all *p *<* *0.0001)

Genetic distance	Linear GD	Logarithmic GD
*D*_est_ (8 microsatellite loci)	0.465	0.630
*F*_ST_ (8 microsatellite loci)	0.369	0.408
*Φ*_ST_ (*rag1*)	0.481	0.530
*Φ*_ST_ (*nad1*)	0.336	0.630

### 3.4 Population size change

The DIYABC analysis of the microsatellite data suggested that a slight reduction in population occurred thousands of generations ago in local populations (Supporting Information Table S6). Four sites were combined into two, 1 + 2 and 3 + 4, given the nonsignificant *F*_ST_ and short geographic distances. For each population, all available loci (8 to 14, mean 11) were used. Fairly consistent results of scenario choice were produced by the direct and logistic regression approaches. The one‐decline scenario resulted in the highest probabilities in 21 (95.5%) and 19 (86.4%) of the 22 populations in the direct and logistic regression approaches, respectively. In the other cases, the growth‐after‐decline scenario resulted in the highest probabilities for population 3 + 4 for both approaches and populations 5 and 22 for the logistic regression approach. In population 3 + 4, if the growth‐after‐decline scenario was not considered, the most likely scenario would have been distinct growth ~3.6 thousand generations ago. It was not surprising that the one‐change scenario was not the most likely in any comparisons because all the special cases of it were covered in other scenarios. However, demographic parameter estimates under the one‐change scenario and the most likely scenario were highly consistent, suggesting that the data had sufficient information to distinguish between these simple scenarios (Supporting Information Table S6). On the other hand, the estimated confidence in scenario choice among the one‐growth, constant‐size, one‐decline, and growth‐after‐decline scenarios was not high. The mean error rate of the direct approach was 0.429, with a mean of 46.3% cases of the best scenario for the observed data being falsely selected for the pseudo‐observed test data and a mean of 30.7% cases of the best scenario being falsely considered as suboptimal. For the logistic regression approach, the corresponding values were 0.427%, 40.8%, and 34.4%, respectively. These evaluations, although they might be influenced by the number of scenarios compared (Cabrera & Palsbøll, 2017), implied that the information was not sufficient for determinable scenario‐comparison and precise parameter estimation in individual populations. Interpreting the results from each population jointly, a decline of 1/4‐fold and three thousand generations ago fitted the data best on average, and the scenarios of a more recent decline or a commonly occurring subsequent growth after decline were not supported.

### 3.5 Species distribution modeling

**Figure 6:**
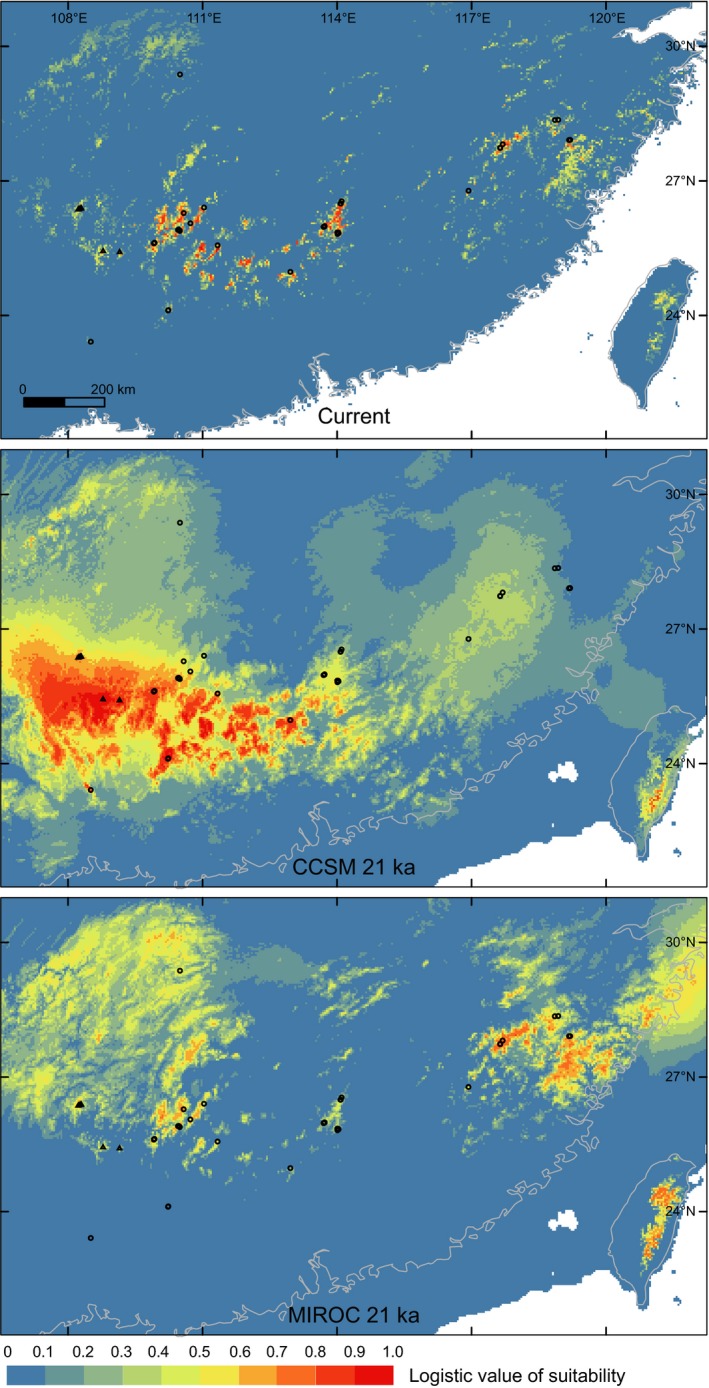
Inferred environmental suitability for *L. liui* + *L. leishanense* determined by Maxent ecological niche modeling. The LGM climate 21,000 years ago was reconstructed using CCSM and MIROC. Circles and triangles are sampling localities

Compared with the current distribution, the inferred distribution of these frogs during the LGM was more continuous and planar. The Maxent model provided reasonable discrimination with high AUC values, with an AUC_training_ of 0.975 ± 0.007 (mean ± *SD*) and an AUC_test_ of 0.971 ± 0.014. Two extreme variables, *T*_war_ (mean temperature of the warmest quarter; 66.8%) and *Prec*_dry_ (precipitation of the driest month; 17.0%), provided the greatest contributions to model development (Supporting Information Table S7). Additionally, the jackknife tests revealed that *T*_war_ was most informative and that *Prec*_dry_ contained the greatest amount of information that the other variables could not provide. Their response curves (Supporting Information Figure S3) indicated that high maximum temperature and low minimum precipitation severely limited the distribution of these cold‐adapted frogs, the tadpoles of which live in permanent streams, which was biologically reasonable. As a result, the present disjunct montane distribution was accurately inferred (Figure 6). These performances suggested that the reliability of the LGM projection would be unlikely to suffer from low reliability of the Maxent model itself. Although the LGM potential distributions based on the CCSM and MIROC reconstructions did not match one another closely, they both overlapped with the major extent of the present distribution and were largely located within the present latitudinal limits (Figure 6). Such a comparison could be obtained using projections in the mainland portion of the study area. In the present mainland, for *T*_war_ and *Prec*_dry_, the only LGM reconstruction outside the training range was a marginally lower minimum precipitation according to MIROC in the most southwestern corner (Supporting Information Figure S3). The response curve showed that the probability of the presence had already declined to near zero at the dry limit of the minimum precipitation range. Therefore, in this case, little effect of the Maxent clamping strategy, treating novel conditions as the same as the limit of the training range, was expected (Phillips, 2017). As *T*_war_ and *Prec*_dry_ contributed 83.8% to model development, we also expected limited, if any, complications caused by novel conditions of the other variables in examining the prediction of a wider and more continuous LGM projection.

## 4 DISCUSSION

Our hypothesis that *L. liui* of western origin responded to Quaternary climatic cycles by spreading from high‐elevation interglacial refugia to more continuous and planar habitats during glacial periods, which allowed eastward dispersals that extended the distribution range to the current one, was not rejected. A closer relationship between *L. leishanense* and nearby *L. liui* than between the latter and the other *L. liui* populations was revealed by PCoA and AMOVA as well as clustering and gene tree analyses of the nuclear microsatellite and *rag1* data (Figures 3 and 5). Given this finding and a lack of morphological diagnostic characters, the predictions of the hypothesis were examined using the results from the combined data of the two putative species. The results are consistent with the predictions and provide details for the hypothesized scenario.

Concordant patterns of sequential eastward divergence were separately observed in the microsatellite clusters and gene trees. The microsatellite clusters and *nad1* lineages corresponding exclusively to regions W_E_, E_W_, and E_E_ diverged eastward from the western W_W_ and W_M_ regions (Figures 1, 3, and 4). A lower level of turnover was presented by the *rag1* tree than by the *nad1* tree (Supporting Information Figure S1). Similar differences between nuclear and mitochondrial loci have frequently been reported and have been explained, for instance, by the smaller effective population size of the mitochondrial marker and male‐mediated gene flow (Knopp & Merila, 2009; Moritz, Dowling, & Brown, 1987). Despite this difference, the same order of divergence was supported by the nesting of *rag1* lineages. The single E_E_ *rag1* haplotype was identical to one of the four E_W_ haplotypes, most of which were nested within one of the three W_E_ lineages, which in turn were nested within a number of W_M_ and W_W_ lineages. The other E_W_ haplotype was nested with a haplotype from site 12, which was located close to W_E_ and showed some admixture of eastern microsatellite alleles as well (Figure 3). The predicted eastward decline in variation during dispersal was detected in all types of genetic data (Figure 1; Supporting Information Table S4). For example, within each of regions W_W_/W_M_, W_E_, E_W_, and E_E_, the average rarefied microsatellite allelic richness across populations was estimated to be 5.86/5.81, 4.60, 3.25, and 2.73, respectively.

The eastward divergences were estimated to have occurred mainly in the Pleistocene (2.588–0.012 Ma) (Figure 4). The mean ages and 95% highest posterior densities of the three sequential *nad1* divergence events were 3.2 (4.1–2.2), 1.0 (1.5–0.6), and 0.4 (0.6–0.2) Ma. The two Pleistocene divergence events, W_E_‐E and then E_W_‐E_E_, could not be assigned to a single glacial phase lasting ~0.1 million years. Therefore, dispersals might have occurred in different glacial periods. Across the distribution, the mean estimates between regional populations ranged from 4.2 to 0.4 Ma, indicating that they have persisted through at least several late Quaternary glacial cycles (~0.1 million years each; Denton et al., 2010).

For the predicted population size decline caused by the contraction after the end of the last glacial period approximately 0.01 Ma, a slight reduction occurring on average three thousand generations ago was inferred for all local populations (Supporting Information Table S6). We acknowledge that the data were insufficient for precise date estimation in individual populations, as the confidence in scenario choice was not high. However, the estimated times (in generations) of the decline for different populations were comparable and, in many cases, similar, suggesting that they were informative. Considering each population as a replicate, these time estimates could be averaged to examine the prediction. Given the generation time of approximately 4 years, the time of the inferred reduction, ~0.012 Ma on average, was comparable to the expected one. Hence, we believe that the result agrees with the prediction.

As predicted, the two‐dimensional IBD model fitted the genetic variation better than the one‐dimensional IBD model across the range (Table 1). In favor of the prediction, with a coefficient of determination (*R* ^2^) up to 0.630, the two‐dimensional IBD pattern detected was fairly strong. Various measures of population differentiation also showed generally high levels of genetic turnover (Supporting Information Table S5). This finding is consistent with the long‐term habitation suggested by the *nad1* time tree and the hypothesized interglacial disjunction (Wielstra et al., 2013).

In general, the inferred distributions during the LGM were more extensive and continuous than the current distribution and were located well within the present latitudinal range. Between the CCSM and MIROC projections, a notable difference was observed around site 22. This local population exhibited exclusively unique sequence variation, suggesting long‐term persistence (Figure 4 and Supporting Information Figure S1). Such independent evidence was consistent with the CCSM projection but inconsistent with the MIROC one, which showed that site 22 and a nearby region would be unsuitable in the LGM (Figure 6). Consequently, the colder and wetter CCSM reconstructions around site 22 might provide a better fit to the two variables that contributed the most to the Maxtent model (Supporting Information Figure S3). In addition to the caveat that these projections should be viewed with caution, this finding again emphasized the various capabilities of different reconstructions when applied to specific parts of the world (e.g., Arenas, Ray, Currat, & Excoffier, 2012; Igea et al., 2013). In recent years, an increasing number of studies have simultaneously used species distribution modeling and molecular and/or fossil data (Gavin et al., 2014). When meta‐analyses of such studies reveal that a climatic variable reconstructed using a particular model is too high/low in a specific region, the finding may be used to help interpret projections and determine model modifications (McGuire & Davis, 2013).

The findings of this study are among the first pieces of evidence that certain cold‐adapted organisms in the subtropical mountains of the East Asian mainland responded to Quaternary climatic oscillations by expanding their ranges during glacials and retreating to high elevations during interglacials. Given this response, the current ranges of these frogs are refugia used for various lengths of time. These ranges overlap extensively with the sampling of previous studies suggesting the same scenario, with Tian et al. (2010) suggesting refugia for pine trees in six mountains (sites 12, 14, 17 + 18, 19, 21, and 23) in the west and Wu et al. (2013) suggesting refugia of salamanders in eight mountains (sites 1 + 2, 5 + 6, 8 + 9, 10, 12, 17 + 18, 21, and 25) across the range. The eastern range (E) was inferred to be established through dispersals that occurred in different Quaternary glacial periods, forming the northeastern limit of the range of this primarily tropical genus of 35 described species (Frost, 2018). In shaping diversity patterns, the contribution of the glacial climate through facilitating the establishment of new distributions for cold‐adapted organisms remains unclear. Modern climate change, on the contrary, is likely a threat to many cold‐adapted species (e.g., Gottfried et al., 2012). Our results support that *L. liui* is isolated in sky‐island refugia by warm low‐elevation habitats. Subtropical East Asia is expected to be warmer in the 21st century (Collins et al., 2013). This trend may lead to habitat reduction and local extinctions of this species, for which the conservation efforts can benefit from the distribution of genetic diversity and evolutionary processes inferred in the present study (Moritz, 2002).

Since this work was submitted for publication, Li et al. (2018) reported a phylogeographic study including 13 *L. liui* and one *L. leishanense* sampling sites and using mitochondrial *cytb* and *nad4* genes and eight microsatellite loci. Based on the time‐calibrated gene tree, the authors proposed that the late Miocene intensification of East Asian monsoons (An et al., 2006; Xia et al., 2009), the warmer climate of the Pliocene (Jiang, 2009; Salzmann et al., 2011), and an early Pleistocene uplift of the Wuyi Mountains (Liu, 1984) shaped the genetic structure of *L. liui*, providing a hypothesis for the causes of its early dispersal and divergence. In addition to molecular dating, they also assessed regional genetic diversity, microsatellite clustering with STRUCTURE, and IBD. The mitochondrial time‐calibrated tree of Li et al. (2018) and the one obtained here had concordant topologies. Although the corresponding nodes were approximately 1.5 times older in the former, the W_E_‐E and subsequent E_W_‐E_E_ splitting events were still dated well within the Pleistocene. In the W_E_ range, a previously unreported population named JXYF was sampled in Li et al. (2018). A comparison of the two trees suggested that the JXYF lineage nested within the W_M_ ones and represented another Pleistocene eastward dispersal event from W_M_ to W_E_. The predicted eastward decline in genetic diversity was also evidenced in Li et al. (2018). However, their analyses did not reveal an eastward divergence of microsatellite clusters and detected a weak IBD pattern in only the microsatellite data. Comparable sequencing and genotyping efforts for individual sampling sites were made in both studies. Compared to our 25 sites, a similar distribution range was covered, but far fewer intermediate populations were included in Li et al. (2018). Thus, a lower level of genetic connectivity of the samples might have contributed to their STRUCTURE result of 11 clusters. For their Mantel correlation tests, the on average larger geographic distances (Rousset, 1997; Whitlock & Mccauley, 1999) and the smaller sample size (*n *=* *13) might have been relevant to the failure to detect a strong IBD pattern across the range of *L. liui*. More generally, these differences again demonstrated the substantial effects of the geographic sampling scheme on spatial genetic structure analyses.

## CONFLICT OF INTEREST

None declared.

## AUTHOR CONTRIBUTIONS

Y.Z. and X.Z. conceived the study. Y.Z. conducted the field and molecular lab work. Y.Z. and J.H. performed the analyses. Y.Z. led the writing with assistance from J.H. and X.Z.

## DATA ACCESSIBILITY

DNA sequences: GenBank accessions KJ484817–KJ485696 and KJ538788–KJ538949. The 14‐loci microsatellite, *nad1*, and *rag1* datasets are available on Dryad Digital Repository: https://doi.org/10.5061/dryad.29kb682.
