# Supplementary material for: Examining the interglacial high‐elevation refugia scenario in East Asian subtropical mountain systems with the frog species Leptobrachium liui
Source: Ecol Evol. 2018 Aug 24;8(18):9326–40. doi: 10.1002/ece3.4449 (PMC6194219; doi:10.1002/ece3.4449)
Supplement: Supplementary file 1 [file ECE3-8-9326-s001.pdf]

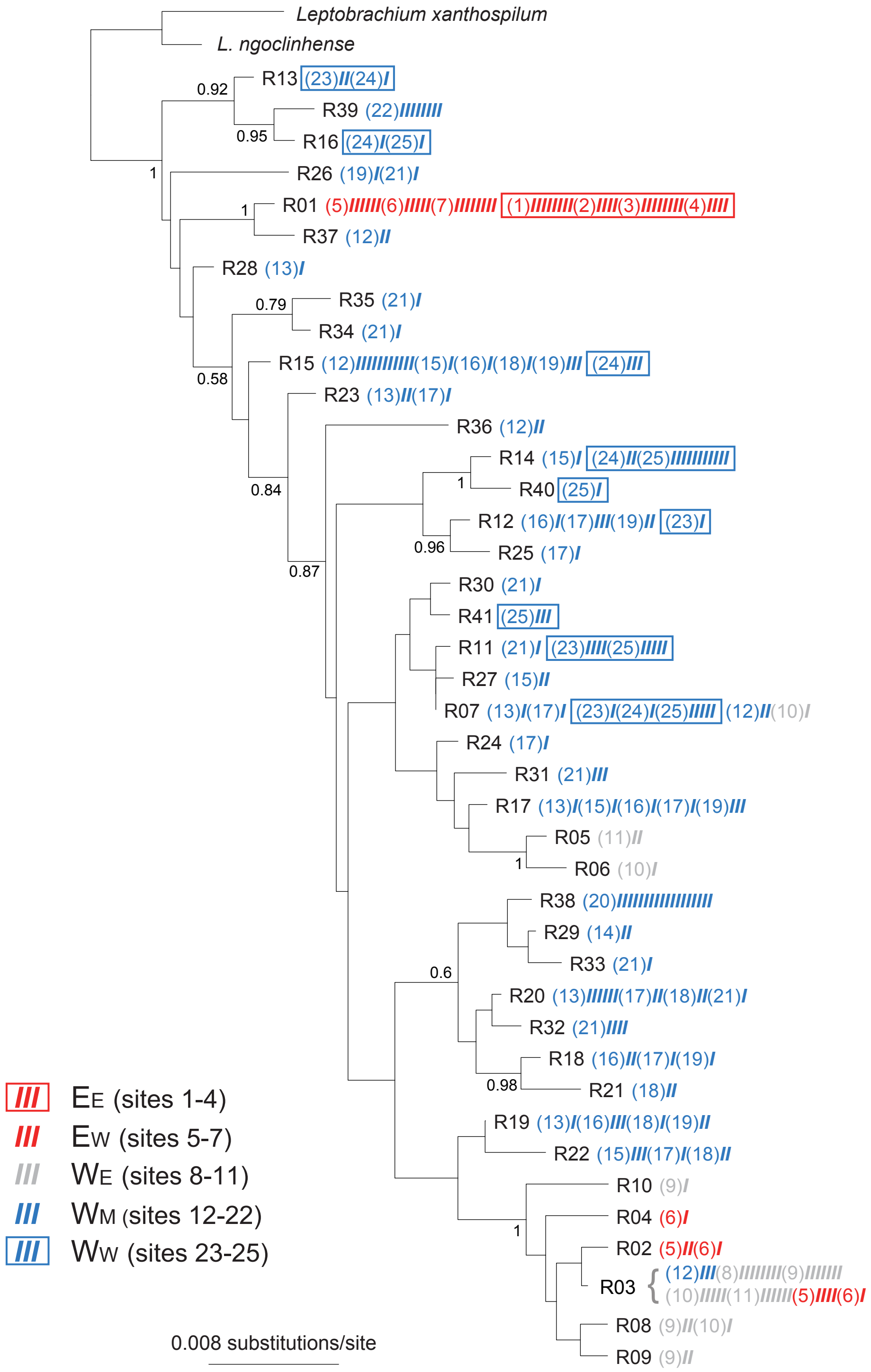

Fig. S1 The *rag1* gene tree of *Leptobrachium liui* and *L. leishanense* obtained by Bayesian inference. The names of the areas and sampling sites correspond with those of Fig. 1. Numbers beside nodes are posterior probabilities ≥ 0.50, and each haplotype is followed by its source sampling sites in brackets and frequency as the number of bars.

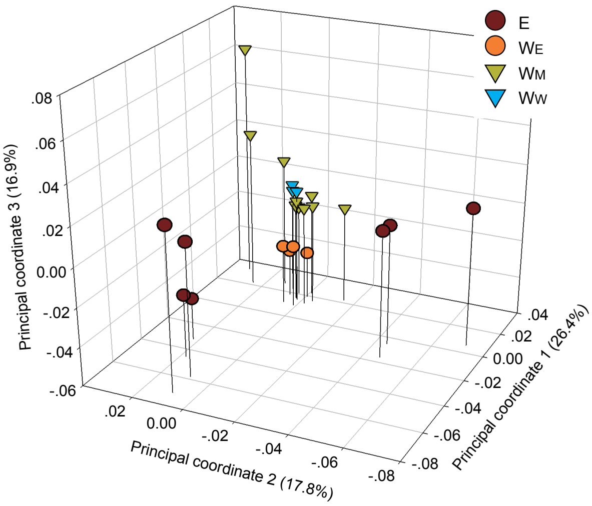

8 microsatellite loci F_{ST}

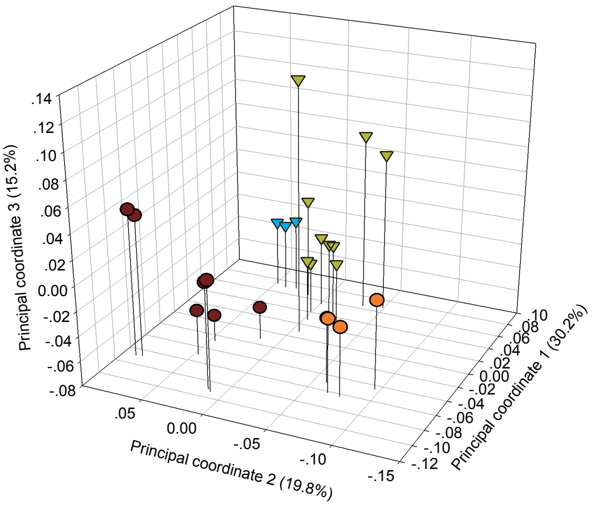

8 microsatellite loci D_{est}

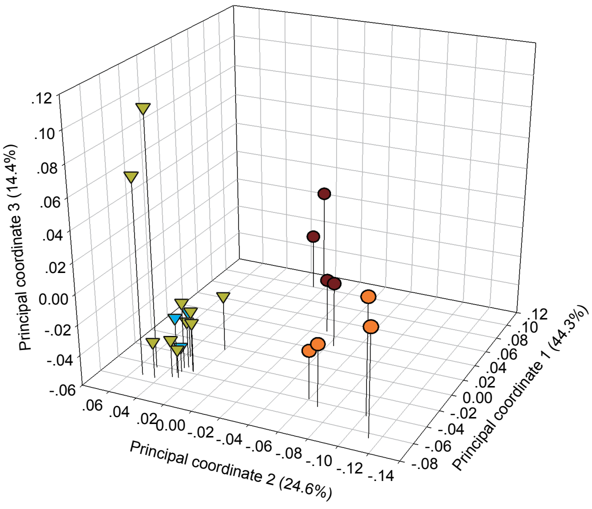

rag1 Φ_{ST}

Fig. S2 Principal coordinates analyses for local populations using F_{ST} and D_{est} estimated from the 8-loci microsatellite data and Φ_{ST} from the *rag1* data. Populations are colored according to their distribution areas.

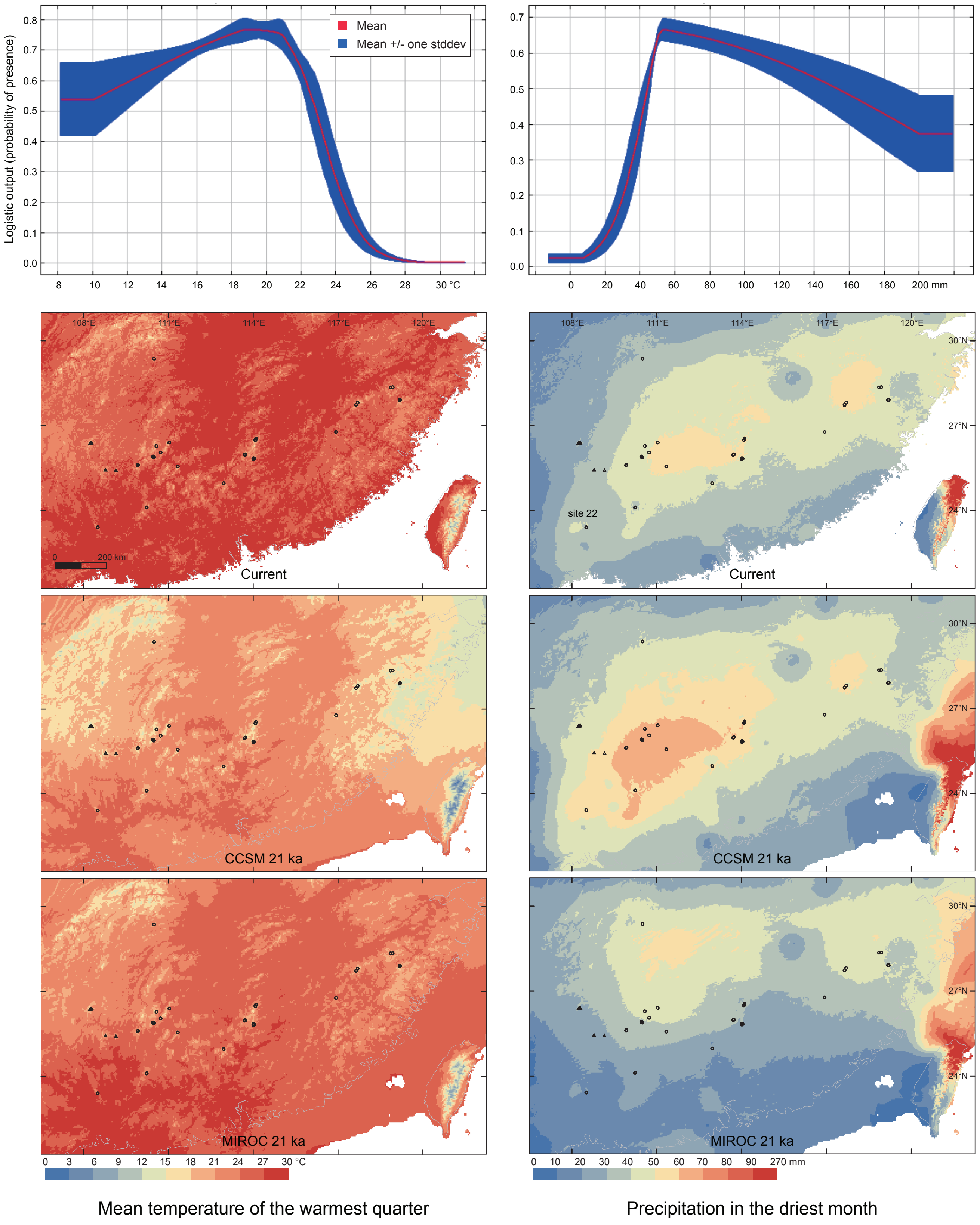

Fig. S3 The response curves (top) and the current estimates and LGM (21 ka) reconstructions of the two variables with the highest contributions to the Maxent model, mean temperature of the warmest quarter (66.8%) and precipitation in the driest month (17.0%). Each response curve was made by using only the corresponding variable. The variables were reconstructed using CCSM and MIROC. Cycles and triangles are sampling localities.
